# 12-month intravascular ultrasound observations from BiOSS® first-in-man studies

**DOI:** 10.1007/s10554-016-0926-9

**Published:** 2016-06-17

**Authors:** Robert J. Gil, Jacek Bil, Ricardo A. Costa, Katarzyna E. Gil, Dobrin Vassiliev

**Affiliations:** 1 Department of Invasive Cardiology, Central Clinical Hospital of the Ministry of Interior, 137 Woloska Street, 02-507 Warsaw, Poland; 2 Institute of Experimental and Clinical Medicine, Polish Academy of Science, Warsaw, Poland; 3 Instituto Dante Pazzanese de Cardiologia, Sao Paulo, Brazil; 4 Alexandrovska University Hospital, Sofia, Bulgaria

**Keywords:** Dedicated bifurcation stent, BiOSS Expert®, BiOSS LIM®, Sirolimus-eluting stent, Paclitaxel-eluting stent, IVUS, QCA

## Abstract

The aim of this study was to analyze the difference in neointima pattern assessed by intravascular ultrasound (IVUS) between two dedicated bifurcation stents, BiOSS® Expert and BiOSS® LIM at 12-month follow-up. This manuscript reports IVUS findings obtained from the analysis of patients enrolled into first-in-man registries initially assessing the BiOSS Expert® (paclitaxel) and BiOSS LIM® (sirolimus) stents. Quantitative angiographic analysis was performed pre, post-stenting, and at follow-up. IVUS examination was performed at 12 months. There were analyzed 34 cases (BiOSS Expert® 11 patients, BiOSS LIM® 23 patients). Procedural characteristics in the two groups were similar, except for rates of main vessel predilatation and FKB/POT, which were higher in BiOSS® LIM group, 54.5 % vs 73.9 % (P < 0.05) and 0 % vs 39.1 % (P < 0.05), respectively. When comparing late lumen loss (LLL) for both stents there were significantly bigger values for main vessel and main branch in the BiOSS® Expert group, but not in side branch. Intravascular ultrasound examination showed that in the BiOSS LIM® group comparing with the BiOSS Expert® group there was lower neointima burden in the whole stent (24.7 ± 7.5 % vs 19.4 ± 8.6 %, P < 0.05) as well as in main vessel (22.8 ± 5.6 % vs 16.9 ± 6.1 %, P < 0.05) and main branch (36.1 ± 6.5 % vs 27.6 ± 8.7 %, P < 0.05), but not at the level of bifurcation (15.1 ± 3.8 % vs 13.6 ± 5.4 %, P = NS). In addition, we found that final kissing balloon/proximal optimization technique (FKB/POT) was associated with significantly smaller value of LLL in main vessel (0.24 ± 0.09 mm vs 0.32 ± 0.14 mm, P < 0.05), which in IVUS analysis resulted in smaller neointima burden in main vessel (13.7 ± 3.9 % vs 18.9 ± 4.45 %, P < 0.05) as well as at the bifurcation site (12.6 ± 4.1 % vs 14.1 ± 2.4 %, P < 0.05). The obtained results suggest that neointima proliferation was the largest in main branches of both stents assessed in quantitative angiography (LLL) as well as in IVUS (neointima burden) and the neointima increase was smaller in BiOSS LIM® stents than in BiOSS Expert® stents. Moreover, the middle part of the stent seems to not to be associated with excessive neointima proliferation and more aggressive protocol of implantation with the use FKB/POT seems to decrease this process.

**Electronic supplementary material:**

The online version of this article (doi:10.1007/s10554-016-0926-9) contains supplementary material, which is available to authorized users.

## Introduction

The bifurcation anatomy varies in terms of the angle, the size of the main vessel/main branch and the side branch, and the underlying plaque distribution, all of which may determine procedural outcomes. The accurate diagnosis of the bifurcation lesion severity and the optimal stent implantation are challenging. Because none of the many current interventional techniques is perfect, dedicated bifurcation stents are under development [1].

Dedicated bifurcation stents are available on the market for several years. Among them there is the BiOSS® stent (Balton, Poland). There are two versions of the BiOSS® stent: paclitaxel-eluting BiOSS® Expert and sirolimus-eluting BiOSS® LIM [2]. Efficacy and safety of these stents have been proved in registries as well as randomized clinical trials [3–8].

Previously, we have analyzed by intravascular ultrasound (IVUS) measurements the mechanisms of lumen enlargement after the coronary bifurcation dedicated stent BiOSS® versus the classical drug eluting stent implantation [9]. Presently, the aim of this study was to analyze the difference in terms of neointima proliferation pattern as assessed by IVUS between BiOSS® Expert and BiOSS® LIM stents at 12 months.

## Methods

### Study population

This manuscript reports the IVUS findings obtained from the analysis of patients enrolled into first-in-man studies initially assessing the BiOSS® Expert and the BiOSS® LIM stents [6, 7]. In both studies inclusion and exclusion criteria were the same. Patients with stable coronary artery disease or non-ST-elevation myocardial infarction were included. The main branch diameter was required to be more than 2.5 mm and the main vessel diameter to be less than 4.5 mm by visual asessment. There was no restriction regarding lesion length and angulation between main branch and side branch. Main exclusion criteria were ST-elevation acute coronary syndrome, bifurcations with Medina 0,0,1, serum creatinine level ≥2.0 mg/dL, inability to take dual antiplatelet therapy for 12 months, bifurcations a priori qualified to the treatment with a two-stent technique as well as the lack of an informed consent. Patients were randomly assigned to IVUS examination of the index stent just before follow-up coronary angiography. Written, informed consent was obtained from all patients before cardiac catheterization.

### Device description

**Fig. 1:**
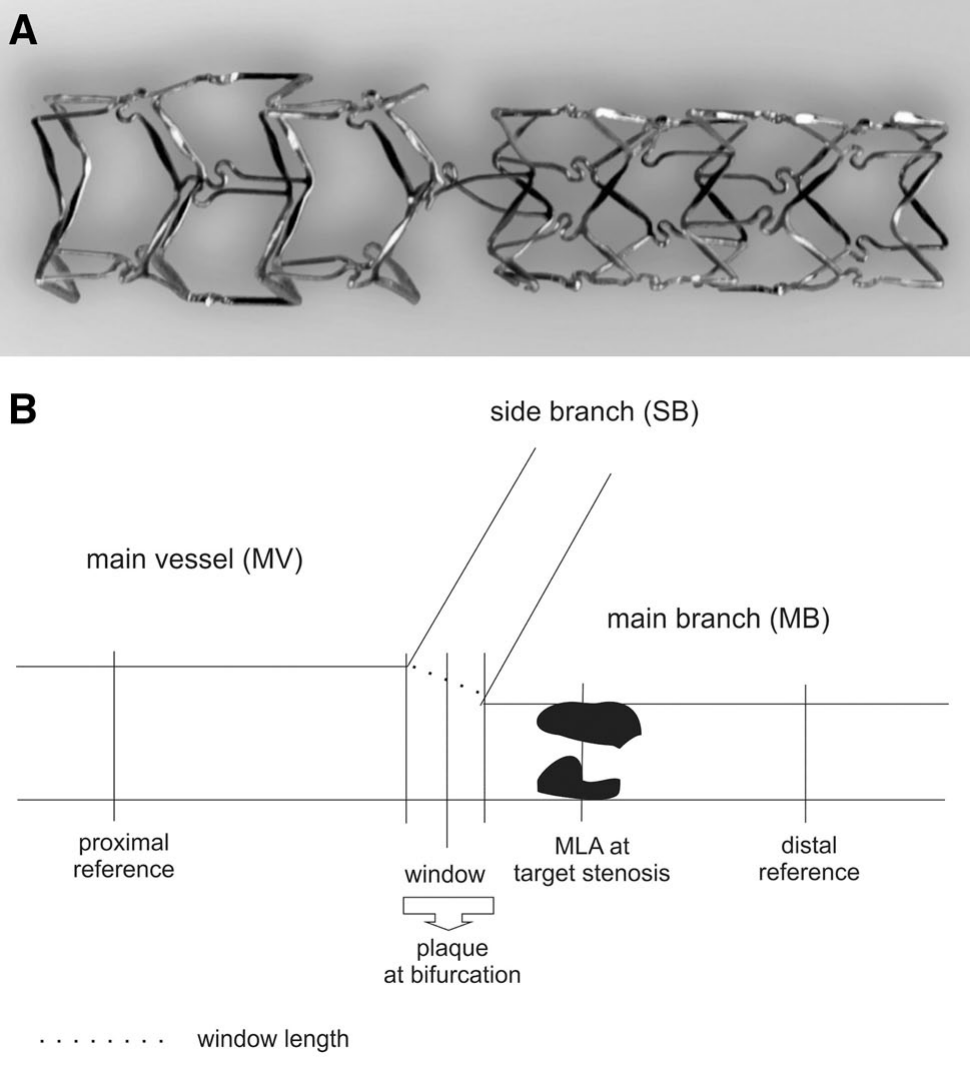
**a** BiOSS® stent structure, **b** the bifurcation structure

The BiOSS® stent, as described previously, consists of two parts, proximal and distal, joined with two connecting struts (depending on size stent 1.9–2.5 mm in length) at the middle zone. The proximal part of the stent has a larger diameter—the proximal/distal diameter ratio is 1.15–1.3. The stent is balloon-expandable and mounted on a stepped delivery Bottle® balloon (Balton, Poland). The BiOSS® stent platform is made of 316L stainless steel (strut thickness 120 µm), and it is a 6-Fr guiding catheter compatible system. It is covered with a mixture of biodegradable poly(lactide-co-glycolide) copolymer and an antiproliferative drug (polymer and drug layer thickness 5 µm). The biodegradation process lasts around 8 weeks. The BiOSS Expert® stent elutes paclitaxel (1 µg/mm^2^) and BiOSS LIM®-sirolimus (1.4 µg/mm^2^) [2] (Fig. 1a).

### Procedure

The detailed procedural details are described elsewhere [6, 7]. Briefly, single stent implantation in the main vessel-main branch across a side branch was the default strategy (provisional T-stenting) in all patients enrolled. There was no restriction regarding lesion length in patient selection. If required, an additional stent was implanted. Proximal optimization technique (POT) and final kissing balloon (FKB) technique were at operator’s discretion. A stent in a side branch was implanted only if proximal residual stenosis was greater than 70 % after balloon dilatation and/or significant flow impairment after main vessel—main branch stenting and/or a flow limiting dissection was present.

### Quantitative coronary angiography analysis

Two orthogonal projections were chosen to visualize the treated bifurcation. All recordings were performed after intracoronary administration of nitroglycerin (200 μg). Quantitative coronary angiographic (QCA) analysis was performed using the dedicated bifurcation software CAAS 5.11 (Pie Medical Imaging BV, the Netherlands). Calibration was performed using the guiding catheter in all cases. Bifurcation lesions were assessed according to Medina classification using an index of one for stenosis greater than 50 and zero for no stenosis (visual estimation) [10]. The three bifurcation segments (main vessel, main branch, side branch) were analyzed separately according to the European Bifurcation Club Consensus (EBC) [11]. The following parameters were reported: lesion length, interpolated reference vessel diameter (RVD), minimal lumen diameter (MLD). Percentage diameter stenosis (%DS), acute lumen gain (ALG) and late lumen loss (LLL) were calculated as described previously [5]. Angiographic success was assessed as the end-procedural main branch diameter stenosis less than 20 % and side branch ostial stenosis less than 70 % without significant dissection and flow impairment.

### Intravascular ultrasound examination

After follow-up coronary angiography, an IVUS catheter (Atlantis SR® or later OptiCross® Coronary Imaging Catheter, Boston Scientific Corporation, Marlborough, MA, USA) was advanced distally to the stented region. Pullback was performed at the speed of 0.5 mm/s until the guiding catheter was reached. External elastic membrane (EEM) was taken as a border of vessel’s total cross-sectional area (vessel area, VA) and was identified as the edge between hypoechoic media and hyperechoic adventitia. Lumen area (LA) was measured by tracing the leading edge of the neointima. Stent area was measured by tracing the edge of the stent. Neointima area was measured as the difference between the value of stent area and the LA. Each of these parameters and both references (proximal and distal) were analyzed in single slices. Neointima burden was calculated according to the formula: (stent area − lumen area)/stent area. The abovementioned parameters were measured at 1 mm intervals. Neointima volume was calculated according to the Simpson’s rule.

IVUS was performed in all cases in the parent vessel (main vessel + main branch) at follow-up. The measurements were performed in the whole stent and in its three crucial parts: main vessel, bifurcation site and main branch. The window length was defined as the largest diameter between carina and vessel wall (or between stent struts) at the level of side branch inflow as seen from the main vessel. The bifurcation site corresponded with the values of three cross-sectional areas: proximal limb, window and distal limb, as described previously [9] (Fig. 1b). Struts in side branch flow were determined as yes (one) or no (zero) value, i.e. one was given when during IVUS examination struts (one or two struts) were present in the side branch window/side branch inflow.

### Statistical analysis

Continuous variables were presented as mean ± SD. Categorical data were presented as numbers (%). Continuous variables were compared using an unpaired Student two-sided *t* test, and categorical data using the χ^2^ test or Fisher exact test, as appropriate. If distribution was not normal (verified with the Shapiro–Wilk test), Wilcoxon signed-rank tests and Mann–Whitney *U* tests were used. P values of <0.05 were considered statistically significant. The inter-observer agreement was tested using Pearson’ s coefficient of correlation. Statistical analyses were performed using R 3.0.2 for OS (R Foundation, Vienna, Austria).

## Results

### Baseline clinical characteristics

Intravascular ultrasound examination at follow-up was performed in 11 (17.5 %) patients with the BiOSS® Expert stent implanted and in 23 (38.3 %) patients with the BiOSS® LIM implanted. Only theses patients were taken into consideration in further analysis. The mean age of patients in the BiOSS® Expert group was 65.1 ± 7.3 years old, and in the BiOSS® LIM group −66.2 ± 6.1 years old (P = NS). In the BiOSS® LIM group there was a significantly higher rate of prior PCI (45.5 % vs 65.2 %, P < 0.05). The detailed characteristics is presented in Table 1. Additionally, in Supplementary Table 1 we presented baseline population characteristics of both BiOSS Expert and BiOSS LIM Registries.

**Table 1:** Baseline population characteristics

Baseline clinical characteristics	BiOSS Expert® group	BiOSS LIM® group
	n = 11 (%)	n = 23 (%)
Age (years)	65.1 ± 7.3	66.2 ± 6.1
Women (%)	3 (27.3)	7 (30.4)
Hypertension	9 (81.8)	19 (82.6)
Hypercholesterolemia	9 (81.8)	17 (73.9)
Diabetes type 2	3 (27.3)	5 (21.7)
Prior MI	4 (36.4)	7 (30.4)
Prior PCI	5 (45.5)	15 (65.2)*
CABG	1 (9.1)	0
Peripheral artery disease	2 (18.2)	3 (13)
Chronic kidney disease	1 (9.1)	1 (4.4)
History of smoking	2 (18.2)	2 (8.8)
EuroScore II (%)	1.21 ± 1.2	1.31 ± 0.9
Clinical indication for PCI		
Planned PCI	10 (91.9)	20 (86.9)
UA	1 (9.1)	2 (8.7)
NSTEMI	0	1 (4.4)
STEMI	0	0

*MI* myocardial infarction, *PCI* percutaneous coronary intervention, *CABG* coronary artery bypass graft, *UA* unstable angina, *NSTEMI* non-ST-elevation myocardial infarction, *STEMI* ST-elevation myocardial infarction

*P < 0.05

### Angiographic and procedural characteristics

The mean SYNTAX score was similar between groups, but in the BiOSS® LIM group there was a higher rate of multivessel disease (54.5 % vs 73.9 %, P < 0.05). In the majority of cases BiOSS® stents were implanted in the left anterior descending artery (BiOSS Expert 63.6 % vs BiOSS LIM 69.6 %, P = NS). Also, rates of true bifurcations were similar between groups (BiOSS® Expert 90.9 % vs BiOSS® LIM 86.9 %, P = NS), but in the BiOSS® LIM group there was a significantly higher rate of true bifurcation type 1, 1, 1 (45.4 % vs 56.5 %, P < 0.05). More details are presented in Table 2.

**Table 2:** Baseline angiographic characteristics

Baseline angiographic characteristics	BiOSS Expert® group	BiOSS LIM® group
	n = 11 (%)	n = 23 (%)
SYNTAX score (points)	16.73 ± 2.44	17.21 ± 3.46
Multivessel disease	6 (54.5)	17 (73.9)*
Lesion type		
A	1 (9.1)	1 (4.4)
B1	6 (54.5)	13 (56.5)
B2	4 (36.4)	8 (34.8)
C	0	1 (4.4)
Lesion location		
LM	3 (27.3)	5 (21.8)
LAD	7 (63.6)	16 (69.6)
LCx	1 (9.1)	1 (4.4)
RCA	0	1 (4.4)
Medina classification		
1,1,1	5 (45.4)	13 (56.5)*
1,0,1	3 (27.3)	5 (21.7)
0,1,1	2 (18.2)	2 (8.7)
1,0,0	0	2 (8.7)
1,1,0	1 (9.1)	1 (4.3)

*LM* left main, *LAD* left anterior descending artery, *LCx* left circumflex artery, *RCA* right coronary artery

*P < 0.05

The main procedural variables are presented in Table 3. Mean BiOSS® Expert stent nominal parameters were as follows: proximal diameter −3.47 ± 0.34 mm, distal diameter −2.85 ± 0.23 mm and length −16.81 ± 1.76 mm, while the mean maximal implantation pressure was 13.4 ± 2.2 atm. In the BiOSS® LIM group those parameters were as follows: proximal diameter −3.54 ± 0.26 mm, distal diameter −2.96 ± 0.43, length −2.96 ± 0.43 mm and mean maximal implantation pressure −12.8 ± 1.9 atm. Procedural characteristics in the two groups were similar, except for rates of main vessel predilatation and final kissing balloon/proximal optimization technique (FKB/POT), which were higher in BiOSS® LIM group, 54.5 % vs 73.9 % (P < 0.05) and 0 % vs 39.1 % (P < 0.05) respectively.

**Table 3:** BiOSS® stent implantation characteristics

Parameter	BiOSS Expert® group	BiOSS LIM® group
	N = 11 (%)	N = 23 (%)
MV predilatation	6 (54.5)	17 (73.9)*
SB predilatation	5 (45.5)	9 (39)
Nominal stent diameter in MV (mm)	3.47 ± 0.34	3.54 ± 0.26
Nominal stent diameter in MB (mm)	2.85 ± 0.23	2.96 ± 0.43
Nominal stent length (mm)	16.81 ± 1.76	17.2 ± 3.2
Mean stent implantation pressure (atm)	13.4 ± 2.2	12.8 ± 1.9
SB postdilatation	3 (27.3)	5 (21.8)
POT	0	9 (39.1)*
FKB	4 (36.3)	8 (34.8)
POT + FKB	0	9 (39.1)*
Additional stent in SB	1 (9.1)	2 (8.7)

*MV* main vessel, *MB* main branch, *SB* side branch, *POT* proximal optimization technique, *FKB* final kissing balloon

*P < 0.05

### Clinical outcomes

During follow-up there was no death or myocardial infarction. In BiOSS® Expert group there were three (27.2 %) cases of target lesion revascularization, and in BiOSS® LIM group two (8.7 %) cases of target lesion revascularization. The detailed data are presented in Supplementary Table 2.

### Quantitative coronary angiography analysis

Angiographic data are presented in Supplementary Table 3. By QCA, baseline in the BiOSS® LIM group there was more severe stenosis in the main branch (40 ± 12 % vs 51 ± 15 %, P < 0.05) and less severe stenosis in the side branch (59 ± 21 % vs 43 ± 13 %, P < 0.05) comparing with the BiOSS® Expert group. The immediate angiographic success rates were 100 % in both groups. Quantitative angiographic analysis revealed that in both groups stent implantation led to a significant increase in MLD and decrease in %DS in the main vessel and in the main branch.

**Fig. 2:**
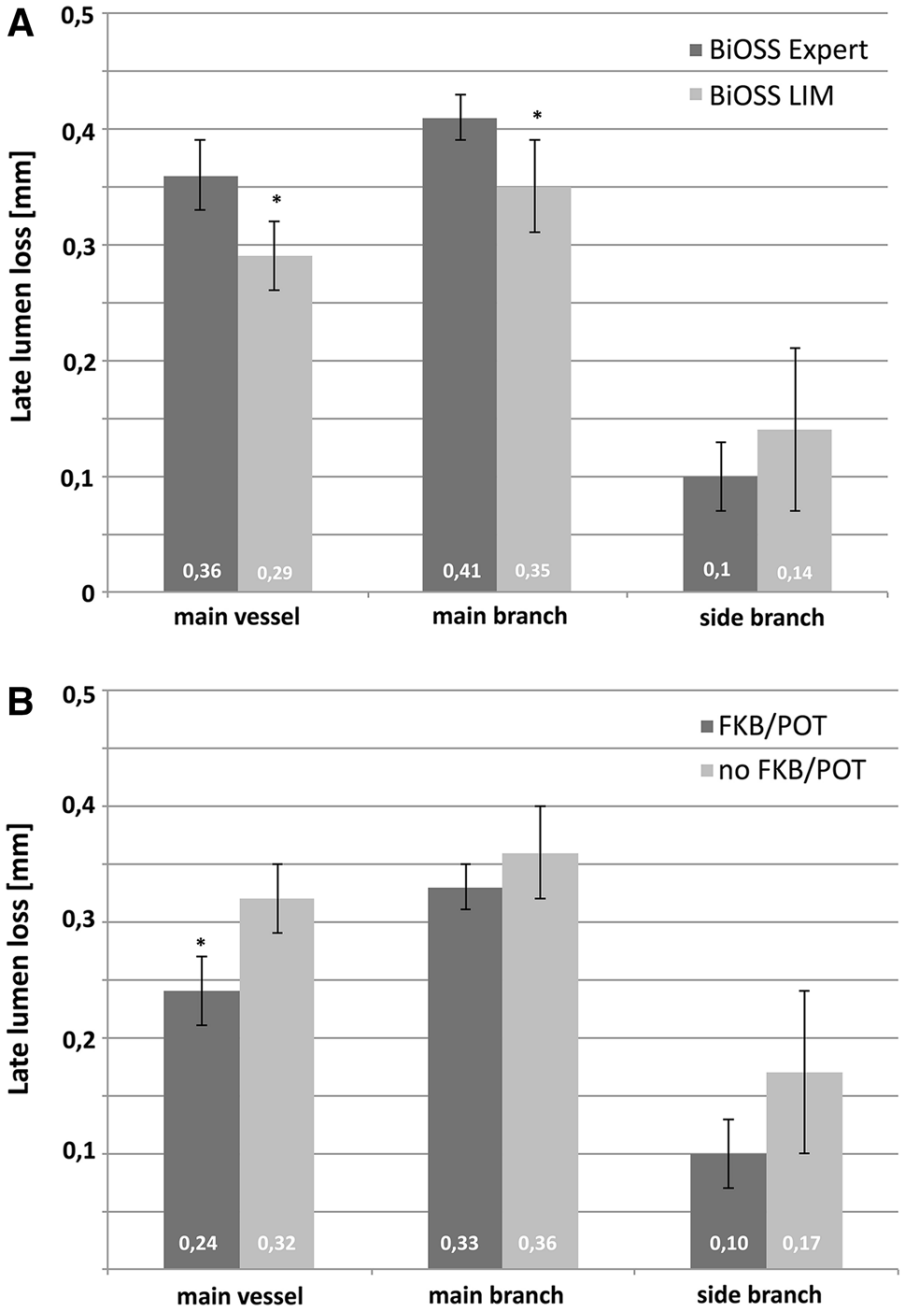
Late lumen loss. **a** The difference between BiOSS Expert® and BiOSS LIM® groups in each bifurcation segment: main vessel, main branch and the side branch; **b** the difference in BiOSS LIM® group depending on optimization technique use with or without FKB/POT. *FKB* final kissing balloon technique, *POT* proximal optimization technique

When comparing LLL values there were significant differences in the main vessel and in the main branch between the BiOSS® Expert and BiOSS® LIM stents, but not in the side branch (Fig. 2). Additionally, when compared the influence of stent optimization (POT/FKB) on LLL values at 12 month follow-up in the BiOSS® LIM group we found that FKB/POT was associated with the significantly smaller value of LLL in the main vessel (0.24 ± 0.09 mm vs 0.32 ± 0.14 mm, P < 0.05). Detailed analysis of the bifurcation site is presented in Supplementary Table 4.

### Intravascular ultrasound analysis

Intravascular ultrasound examination results are presented in Table 4. Among 578 segments the inter-observer agreement was high (r = 0.88, 95 % CI 0.84–0.92, P < 0.05). In the BiOSS® LIM group comparing with the BiOSS® Expert group there was a larger mean LA measured in the whole stent (6.77 ± 0.6 mm vs 7.57 ± 0.7 mm, P < 0.05), as well as in its each segment: the main vessel (8.2 ± 0.8 mm vs 9.01 ± 1.1 mm, P < 0.05), the bifurcation site (7.4 ± 1.1 mm vs 8.1 ± 0.9 mm, P < 0.05) and the main branch (4.7 ± 0.8 mm vs 5.6 ± 1 mm, P < 0.05). This was associated with less neointima burden in the BiOSS® LIM group in the whole stent (24.7 ± 7.5 % vs 19.4 ± 8.6 %, P < 0.05) as well as in the main vessel (22.8 ± 5.6 % vs 16.9 ± 6.1 %, P < 0.05) and the main branch (36.1 ± 6.5 % vs 27.6 ± 8.7 %, P < 0.05), but not at the level of bifurcation (15.1 ± 3.8 % vs 13.6 ± 5.4 %, P = NS).

**Table 4:** Follow-up IVUS measurements comparing BiOSS Expert® and BiOSS LIM® groups

Parameter	BiOSS® Expert	BiOSS® LIM
	N = 11	N = 23
Qualitative IVUS analysis		
Struts in SB inflow (% of cases)	3 (27.3)**	4 (17.4)***
Incomplete stent apposition	0	0
Quantitative IVUS analysis		
Proximal reference lumen area (mm^2^)	10.44 ± 1.87	10.54 ± 1.32
Distal reference lumen area (mm^2^)	6.96 ± 1.56	7.01 ± 1.2
Mean values-whole stent analysis		
Lumen area (mm^2^)	6.77 ± 0.6	7.57 ± 0.7*
Stent area (mm^2^)	8.9 ± 0.5	9.32 ± 0.6
Vessel area (mm^2^)	17.16 ± 1.2	17.88 ± 1
Neointima area (mm^2^)	2.13 ± 0.65	1.75 ± 0.7*
Neointima burden (%)	24.7 ± 7.5	19.4 ± 8.6*
Neointima volume (mL)	35.78 ± 9.4	29.4 ± 12
Mean values-MV part		
Lumen area (mm^2^)	8.2 ± 0.8	9.01 ± 1.1*
Stent area (mm^2^)	10.62 ± 0.7	10.84 ± 1
Vessel area (mm^2^)	18.46 ± 1.1	19.23 ± 1.3
Neointima area (mm^2^)	2.42 ± 0.9	1.83 ± 0.5*
Neointima burden (%)	22.8 ± 5.6	16.9 ± 6.1*
Neointima volume (mL)	12.8 ± 5.1	9.7 ± 3.8*
Distance between MLA and bifurcation site (mm)	4.2 ± 1.2	3.9 ± 1.5
Mean values-bifurcation site		
Window length (mm)	2.18 ± 0.27	2.24 ± 0.21
Lumen area (mm^2^)	7.4 ± 1.1	8.1 ± 0.9*
Stent area (mm^2^)	8.7 ± 1.3	9.37 ± 0.4*
Vessel area (mm^2^)	17.7 ± 4.1	18.64 ± 3.2
Neointima area (mm^2^)	1.31 ± 0.5	1.27 ± 0.3
Neointima burden (%)	15.1 ± 3.8	13.6 ± 5.4
Neointima volume (mL)	5.07 ± 1.7	4.9 ± 2.1
Mean values-MB part		
Lumen area (mm^2^)	4.7 ± 0.8	5.6 ± 1*
Stent area (mm^2^)	7.35 ± 1.3	7.74 ± 1*
Vessel area (mm^2^)	15.3 ± 0.8	15.78 ± 1
Neointima area (mm^2^)	2.65 ± 1	2.14 ± 0.7*
Neointima burden (%)	36.1 ± 6.5	27.6 ± 8.7*
Neointima volume (mL)	17.9 ± 4.2	14.4 ± 5.4
Distance between MLA and bifurcation site (mm)	4.8 ± 0.9	5.3 ± 2.1

*MV* main vessel, *MB* main branch, *MLA* minimal lumen area

*P < 0.05

**All cases without POT/FKB

***Three cases without POT/FKB

**Fig. 3:**
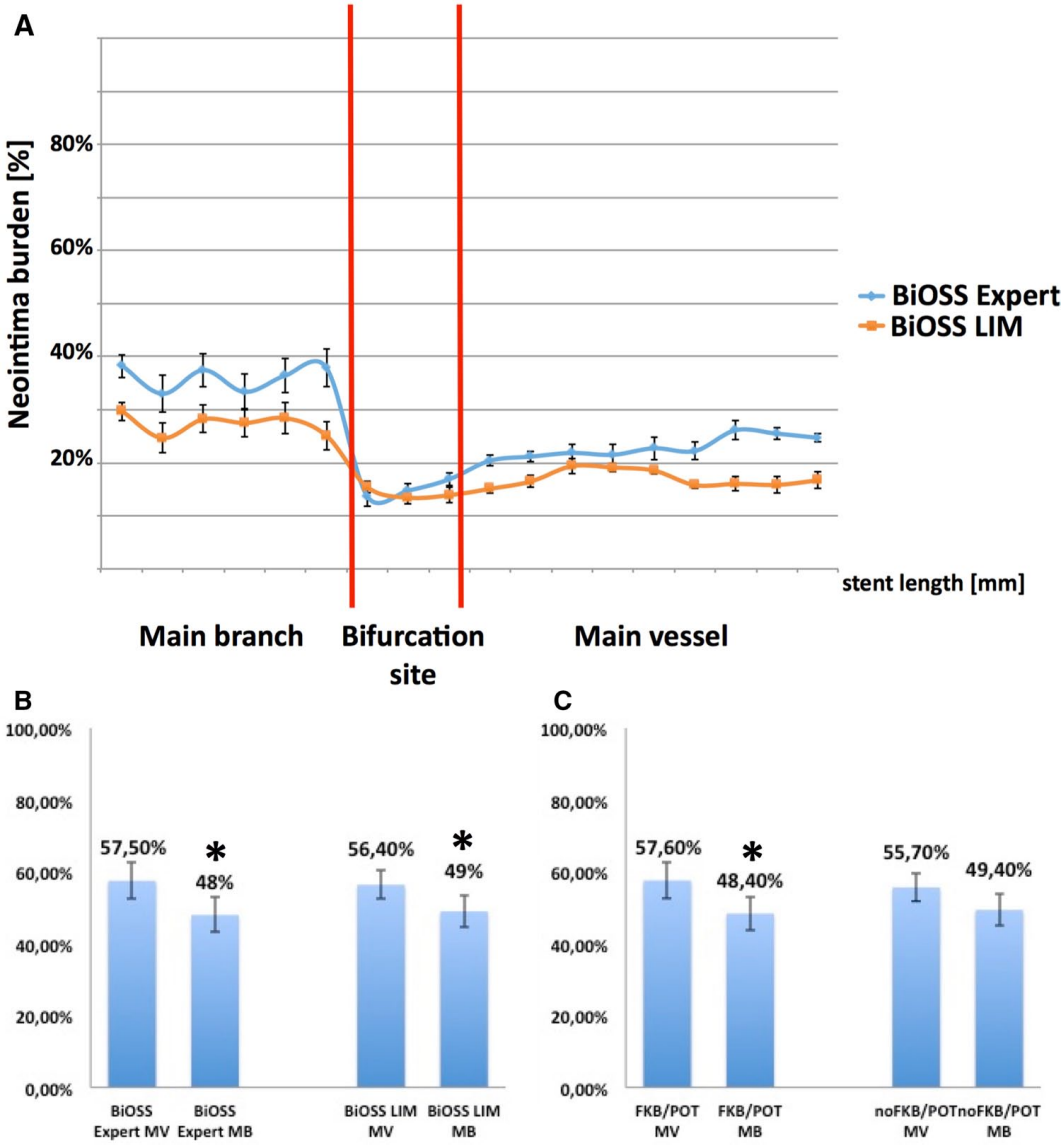
IVUS analysis. **a** The change of neointima burden in particular parts of the analyzed stents BiOSS LIM and BiOSS Expert, **b** the ratio of stent area to vessel area in the main vessel and main branch in BiOSS Expert and BiOSS LIM, **c** the ratio of stent area to vessel area in the main vessel and main branch in the BiOSS LIM in FKB/POT subgroup vs no FKB/POT subgroup

When compared the main vessel, the main branch and the bifurcation site in each group separately (BiOSS® Expert vs BiOSS® LIM), in both of them the biggest neointima burden was observed in the main branch (36.1 ± 6.5 % vs 27.6 ± 8.7 %, P < 0.05), and the smallest at the bifurcation site (15.1 ± 3.8 % vs 13.6 ± 5.4 %, P = NS) (Fig. 3a).

Additionally, to assess stent expansion we calculated the ratio of stent area to vessel area. The mean values in both groups were significantly bigger in the main vessel compared with the main branch. The significance disappeared for the BiOSS LIM® subgroup treated without FKB/POT (Fig. 3b, c).

Similarly to QCA, when compared the influence of stent optimization techniques (POT/FKB) on vessel lumen at the follow-up we found that FKB/POT was associated with the significantly larger LA at the follow-up in the main vessel (9.5 ± 0.9 mm vs 8.7 ± 0.8 mm, P < 0.05) and smaller neointima burden in the main vessel (13.7 ± 3.9 % vs 18.9 ± 4.45 %, P < 0.05) as well as at the bifurcation site (12.6 ± 4.1 % vs 14.1 ± 2.4 %, P < 0.05). The detailed data are presented in Table 5.

**Table 5:** IVUS analysis regarding optimization techniques in BiOSS® LIM subgroup

Parameter	With FKB/POT			Without FKB/POT		
	MV	Bifurcation site	MB	MV	Bifurcation site	MB
Lumen area (mm^2^)	9.5 ± 0.9	8.3 ± 0.8	5.8 ± 1.1	8.7 ± 0.8*	7.9 ± 0.9	5.5 ± 1.3
Stent area (mm^2^)	11 ± 0.8	9.5 ± 0.7	7.8 ± 0.9	10.7 ± 0.7	9.3 ± 0.9	7.7 ± 1.1
Vessel area (mm^2^)	19.1 ± 1.5	18.7 ± 2.3	16.1 ± 1.9	19.2 ± 2	18.6 ± 1.7	15.6 ± 1.9
Neointima area (mm^2^)	1.5 ± 0.4	1.2 ± 0.4	2.1 ± 0.3	2 ± 0.3*	1.3 ± 0.2	2.2 ± 0.5
Neointima burden (%)	13.7 ± 3.9	12.6 ± 4.1	26.5 ± 5.1	18.9 ± 4.4*	14.1 ± 2.4*	28.3 ± 2.5
Neointima volume (mL)	9.2 ± 1.5	4.4 ± 1.2	14.1 ± 3.8	10 ± 1.8*	5.2 ± 1.1*	14.7 ± 2.2
Window length (mm)	–	2.05 ± 0.18	–	–	2.36 ± 0.23*	–

*MV* main vessel, *MB* main branch, *SB* side branch, *FKB* final kissing balloon technique, *POT* proximal optimization technique

*P < 0.05

## Discussion

The main findings of this study are as follows: (1) the neointima increase was significantly larger in the main branches of both examined stents as assessed in QCA (late lumen loss) as well as in IVUS (neointima burden), (2) the middle part of the stent was not associated with the excessive neointima proliferation, (3) the neointima increase was significantly smaller in BiOSS® LIM stents than in BiOSS® Expert stents, and 94) optimization techniques in case of BiOSS® LIM stents greatly improved the angiographic and IVUS outcomes.

On the market there are two versions of the BiOSS® stent: the BiOSS® Expert eluting paclitaxel and BiOSS® LIM eluting sirolimus. In our study both stents were assessed. These two populations did not differ significantly regarding baseline characteristics of patients nor lesions. But consequently the BiOSS® LIM stent proved to be superior comparing with the BiOSS® Expert stent taking into consideration both angiographic as well as IVUS parameters. The mean LLL was significantly lower in the BiOSS® LIM group than in the BIOSS® Expert group, 0.32 ± 0.11 mm vs 0.39 ± 0.14 mm (P < 0.05), respectively. Similarly, the neointima burden was larger in the BiOSS® Expert group, 24.7 ± 7.5 % vs 19.4 ± 8.6 % (P < 0.05), respectively.

The analysis of clinical outcomes in the studied population seems to confirm their association with the neointimal proliferation. Although, probably due to the small number of patients there was only a trend in favor of the BiOSS® LIM stent [TLR 3/11 (27.2 %) vs 2/23 (8.7 %), P = 0.15], but in the whole registry population of the BiOSS Expert® stent the 12-month TLR rate was 11.3 % (7/63), whereas in the BiOSS LIM® registry it reached 8.3 % (5/60), P = 005 [6, 7].

The obtained results are concert with those in previous papers showing that sirolimus-eluting stents (SES) are better than paclitaxel-eluting ones (PES) in terms of efficacy in the bifurcation treatment [12]. In paper Chen et al. after 12-month follow-up results in paclitaxel group differed significantly with sirolimus group regarding to the rate of TLR, TVR and MACE, 12.2 % vs 3.2 % (P = 0.006), 14.4 % vs 4.9 % (P = 0.02) and 20 % vs 10.3 % (P = 0.04), respectively [13]. However, on contrary to us, no control angiography was planned after 12 months. Moreover, Song et al. published paper comparing PES and SES in bifurcation stenting [14]. After 2 years of follow-up in PES group the rate of MACE was 28.6 % and in SES 10.6 % (P = 0.03), whereas the LLL was 1.03 ± 0.45 and 0.28 ± 0.54 mm (P < 0.001) respectively. Also, in a meta-analysis it was proved that when comparing with PES, SES reduced the incidence of TLR, main-branch restenosis and MACE in coronary bifurcation intervention, while the risk of stent thrombosis was similar between SES and PES groups [15].

In our previous paper, the successful BiOSS® Expert stent implantation caused significant increase in LA in each part of the bifurcation: the main vessel, the bifurcation site as well as in the main branch. Actually, the only significant difference between conventional drug-eluting stents vs BiOSS® stents after intervention was found for the window length, which was significantly longer in the group where the BiOSS® stent was implanted (P = 0.01) [9].

After 12 months, the window length was comparable as just after stenting. In the follow-up the window length in the BiOSS® Expert group was 2.18 ± 0.27 mm (value just after stenting 2.21 ± 0.37 mm, P = NS). The neointima proliferation was larger in the main branch comparing with the main vessel. This was true both for the BiOSS® Expert and for the BiOSS® LIM stents. The Fig. 2a shows LLL (main vessel 0.36 ± 0.14 vs 0.29 ± 0.11, main branch 0.41 ± 0.15 vs 0.35 ± 0.12 mm). Moreover, in the Fig. 3 there is presented neointima burden in three parts of the stent. Similarly, in the BiOSS Expert® group there is higher neointima proliferation in the main vessel (22.8 ± 5.6 % vs 16.9 ± 6.1 %, P < 0.05) and in the main branch (36.1 ± 6.5 % vs 27.6 ± 8.7 %, P < 0.05) than in the BiOSS® LIM group. There was no difference between stents at the bifurcation site where there are only two stent struts (15.1 ± 3.8 % vs 13.6 ± 5.4 %, P = NS). Worth mentioning is the fact that this area (bifurcation site) is the region with the smallest neointima burden in the whole stent. Also, in IVUS examination the location of minimal LA site was quite remote from the bifurcation site. Interestingly, there were fewer struts in SB inflow with BiOSS LIM, probably due to higher performance of FKB/POT in this group compared to BiOSS Expert.

The larger neointima proliferation in the main branch might be explained by the smaller diameter of this part of the vessel. And it was proven that in stents with smaller diameter there is a higher risk of neointima proliferation and cardiovascular events [16]. However, we were searching for a much more precise explanation. Measurements of vessel, lumen and plaque areas before and after stenting created an opportunity to identify mechanisms of the lumen enlargement at regions of interest including vessel extension (stretch) and plaque re-distribution. This first mechanism (more stimulating neointimal proliferation) overweighted in distal limb (means distal branch) while in proximal limb (main vessel) and in the mid zone (carina region) this mechanism was less pronounced (43 % vs 46 %, respectively main branch and mid zone) [9]. This less traumatic mechanism of lumen enlargement somehow is responsible for relatively small neointimal proliferation at these levels, however one must remember that additional optimization (FKB/POT) was not performed in that population. Nevertheless, it is pretty sure that vessel expansion would not reach an excessive degree, especially with a dedicated bifurcation device such as BiOSS, which is built on a metallic platform with different diameters at proximal (larger) and distal (smaller) parts in order to optimize scaffold and expansion at the bifurcation anatomy, while maintaining side branch patency.

Additionally, two more factors have the influence on the intima proliferation: proper stent size selection and correct stent strut apposition. In our study in the IVUS analysis the proximal and distal parts of the BiOSS® stents were well apposed. But the distal part of the stent was not optimized during the implantation procedure. FKB/POT optimized the proximal part of the stent only. Additional analysis of the mean ratio of stent area to vessel area calculated for main vessels and main branches in both groups allowed us to assess stent expansion. Obtained results showed that sizing was more proper in case of the main vessel and proved the crucial role of FKB/POT for better outcomes (Fig. 3b, c). Still, one would consider that the amount of plaque along with its distribution within the bifurcation segments (higher amount in the MB) at baseline could explain these findings, at least to certain extent; however, this hypothesis remains purely speculative, as IVUS was not systematically performed at baseline.

FKB inflation technique as well as proxi POT mal optimization technique are the two most commonly recommended by the European Bifurcation Club [17]. Since there were no POT cases in the BiOSS® Expert implantations, we analyzed the influence of these two techniques only in the BiOSS® LIM group. Indeed, we found that in the BiOSS® LIM the neointima proliferation in the main vessel and at the bifurcation site was significantly lower in the group were FKB/POT was applied (Fig. 2b; Table 5). There was no difference in the neointima proliferation in the main branch between these two groups. It seems that optimization techniques are crucial for good results of the BiOSS® stent implantation and ensure the low neointima proliferation.

These results are in agreement with our previous clinical trial, POLBOS I, in which application of FKB and POT was associated with lower LLL and better clinical outcomes [4]. These findings were confirmed in other studies, also in trials with other dedicated bifurcation stents [18–20].

### Study limitations

This study has also some limitations. The number of treated patients that underwent IVUS examination at late follow-up was small and they were selected by operators based on operator’s skills and the imaging catheter availability. Bifurcation lesions a priori qualified to the treatment with a two-stent technique were excluded. Also, no uniform implant technique was used, however procedures were performed by operators highly experienced in BiOSS® stent implantation. And additionally, no control group was introduced to compare the use of this dedicated bifurcation stent and stenting with other devices and techniques.

## Conclusions

The obtained results suggest that neointima proliferation was the largest in main branches of both stents assessed in quantitative angiography (LLL) as well as in IVUS (neointima burden) and the neointima increase was smaller in BiOSS LIM® stents than in BiOSS Expert® stents. Moreover, the middle part of the stent seems to not to be associated with excessive neointima proliferation and more aggressive protocol of implantation with the use FKB/POT seems to decrease this process.

## Electronic supplementary material

Below is the link to the electronic supplementary material.

Supplementary material 1 (DOCX 33 KB)
