# Supplementary material for: 12-month intravascular ultrasound observations from BiOSS® first-in-man studies
Source: Int J Cardiovasc Imaging. 2016 Jun 17;32(9):1339–47. doi: 10.1007/s10554-016-0926-9 (PMC5067288; doi:10.1007/s10554-016-0926-9)
Supplement: Supplementary file 1 — Supplementary material 1 (DOCX 33 KB) [file 10554_2016_926_MOESM1_ESM.docx]

**Supplementary Table 1**. Baseline population characteristics of whole BiOSS Expert® and BiOSS LIM® Registries

|  | | **BiOSS Expert^®^ Registry** | **BiOSS LIM^®^ Registry** |
| --- | --- | --- | --- |
| **Baseline clinical characteristics** | | **n = 63 (%)** | **n = 60 (%)** |
| Age [years] | | 67 ± 11 | 66.4 ± 11 |
| Women [%] | | 25 (40) | 17 (28.3) |
| Hypertension | | 35 (56) | 47 (78) |
| Hypercholesterolemia | | 24 (38) | 47 (78) |
| Diabetes type 2 | | 17 (27) | 23 (38) |
| Prior MI | | 19 (30) | 17 (28) |
| Prior PCI | | 24 (38) | 28 (46.7) |
| CABG | | 6 (10) | 6 (10) |
| Peripheral artery disease | | 12 (19) | 5 (8) |
| Chronic kidney disease | | 4 (6.3) | 10 (16.7) |
| History of smoking | | 11 (17.4) | 20 (33.3) |
| EuroScore II [%] | | 1.1 ± 1.1% | 1.21 ± 0.8% |
| Clinical indication for PCI | |  |  |
|  | planned PCI | 31 (49) | 47 (78.3) |
|  | UA | 20 (32) | 10 (16.7) |
|  | NSTEMI | 12 (19) | 3 (5) |
|  | STEMI | 0 | 0 |

MI – myocardial infarction, PCI – percutaneous coronary intervention, CABG – coronary artery bypass graft, UA – unstable angina, NSTEMI – non-ST-elevation myocardial infarction, STEMI – ST-elevation myocardial infarction; *P < 0.05

**Supplementary Table 2.** Clinical results

|  | **BiOSS Expert^®^ Group**  **n = 11 (%)** | |  | **BiOSS LIM^®^ Group**  **n = 23 (%)** | |
| --- | --- | --- | --- | --- | --- |
|  | **30 days** | **12 mo** |  | **30 days** | **12 mo** |
| **cardiac death** | 0 | 0 |  | 0 | 0 |
| **MI** | 0 | 0 |  | 0 | 0 |
| **Definite ST** | 0 | 0 |  | 0 | 0 |
| **TLR** | 0 | 3 (27.2) |  | 0 | 2 (8.7) |
| **TVR** | 0 | 4 (36.4) |  | 0 | 3 (13) |

MI – myocardial infarction, ST – stent thrombosis, TLR – target lesion revascularization, TVR – target vessel revascularization,

.

**Supplementary Table 3.** Quantitative coronary angiography data

|  | **BiOSS**^®^ **Expert Group** | **BiOSS**^®^ **LIM Group** |
| --- | --- | --- |
| **Preintervention** | | |
| MV – RVD [mm] | 3.54±0.13 | 3.59±0.24 |
| MV - %DS | 64±18 | 60±21 |
| MV MLD [mm] | 1.27±0.16 | 1.44±0.21 |
| MB – RVD [mm] | 2.85±0.1 | 2.94±0.19 |
| MB - %DS | 40±12 | 51±15* |
| MB MLD [mm] | 1.71±0.15 | 1.44±0.11* |
| SB – RVD [mmm] | 2.5±0.12 | 2.29±0.26* |
| SB - %DS | 59±21 | 43±13* |
| SB MLD [mm] | 1.03±0.1 | 1.31±0.17* |
| MV lesion length [mm] | 8.2±2.9 | 9.3±3.5 |
| MB lesion length [mm] | 7.9±2.4 | 8.6±2.9 |
| SB lesion length [mm] | 3.2±2.3 | 4.9±3.1 |
| **Postintervention** | | |
| MV – RVD [mm] | 3.51±0.11 | 3.57±0.15 |
| MV - %DS | 9±3 | 11±4 |
| MV MLD [mm] | 3.17±0.14 | 3.19±0.17 |
| MV ALG [mm] | 1.9 ± 0.21 | 1.75 ± 0.14 |
| MB – RVD [mm] | 2.9±0.11 | 2.99±0.15 |
| MB - %DS | 13±9 | 15±7 |
| MB MLD [mm] | 2.52±0.18 | 2.54±0.11 |
| MB ALG [mm] | 0.81 ± 0.26 | 1.1 ± 0.13* |
| SB – RVD [mmm] | 2.4±0.15 | 2.5±0.3 |
| SB - %DS | 42±15 | 36±19 |
| SB MLD [mm] | 1.39±0.18 | 1.6±0.19 |
| SB ALG [mm] | 0.36 ± 0.09 | 0.39 ± 0.12 |
| **Follow up** | | |
| MV – RVD [mm] | 3.5±0.11 | 3.55±0.13 |
| MV - %DS | 19.7±6 | 18±3 |
| MV MLD [mm] | 2.81±0.15 | 2.9±0.19 |
| MB – RVD [mm] | 2.91±0.08 | 2.94±0.09 |
| MB - %DS | 27.5±13 | 25.5±16 |
| MB MLD [mm] | 2.11±0.26 | 2.19±0.18 |
| SB – RVD [mmm] | 2.45±0.1 | 2.52±0.21 |
| SB - %DS | 47.3±12 | 42.1±16 |
| SB MLD [mm] | 1.29±0.1 | 1.46±0.13* |

MV – main vessel, MB – main branch, SB – side branch, RVD – reference vessel diameter, %DS -- % diameter stenosis, MLD – minimal lumen diameter, ALG – acute lumen gain

**Supplementary Table 4.** Detailed IVUS analysis of bifurcation site

|  | **BiOSS Expert Group** | **BiOSS LIM Group** |
| --- | --- | --- |
| **Proximal limb** | | |
| - lumen area [mm^2^] | 7.8 ± 0.9 | 8.5 ± 1.2* |
| - stent area [mm^2^] | 9.1 ± 1.3 | 9.8 ± 0.7* |
| - vessel area [mm^2^] | 18.0 ± 4.3 | 19.0 ± 2.9 |
| - neointima area [mm^2^] | 1.23 ± 0.4 | 1.2 ± 0.6 |
| - neointima burden [%] | 13.5 ± 3.9 | 12.2 ± 3.8 |
| - neointima volume [mL] | 4.7 ± 2.1 | 4.5 ± 1.8 |
| **Window** | | |
| - window length [mm] | 2.18 ± 0.27 | 2.24 ± 0.21 |
| - lumen area [mm^2^] | 7.6 ± 0.8 | 8.3 ± 1.1 |
| - stent area [mm^2^] | 9 ± 1.4 | 9.7 ± 1.2 |
| - vessel area [mm^2^] | 17.9 ± 5.4 | 18.9 ± 5.1 |
| - neointima area [mm^2^] | 1.29 ± 0.3 | 1.3 ± 0.4 |
| - neointima burden [%] | 14.3 ± 2.6 | 12.9 ± 3.2 |
| - neointima volume [mL] | 4.9 ± 1.9 | 4.7 ± 1.6 |
| **Distal limb** | | |
| - lumen area [mm^2^] | 6.8 ± 0.7 | 7.4 ± 1.2 |
| - stent area [mm^2^] | 8 ± 0.9 | 8.6 ± 1.1 |
| - vessel area [mm^2^] | 17.3 ± 4.5 | 18.2 ± 3.4 |
| - neointima area [mm^2^] | 1.41 ± 0.3 | 1.4 ± 0.2 |
| - neointima burden [%] | 17.6 ± 4.3 | 15.9 ± 3.7 |
| - neointima volume [mL] | 5.6 ± 3.3 | 5.4 ± 3.9 |
